# Differences in the Anthropometric and Physiological Profiles of Hungarian Male Rowers of Various Age Categories, Rankings and Career Lengths: Selection Problems

**DOI:** 10.3389/fphys.2021.747781

**Published:** 2021-10-13

**Authors:** Zoltan Alfőldi, Krzysztof Borysławski, Ferenc Ihasz, Imre Soós, Robert Podstawski

**Affiliations:** ^1^Doctoral School of Health Sciences, Faculty of Health Sciences, University of Pécs, Pécs, Hungary; ^2^Institute of Health, Angelus Silesius State University, Wałbrzych, Poland; ^3^Faculty of Psychology and Pedagogy, Institute of Sports Sciences, Eötvös Lóránd University, Szombathely, Hungary; ^4^Department of Tourism, Recreation and Ecology, University of Warmia and Mazury in Olsztyn, Olsztyn, Poland

**Keywords:** rowing, age categories, ranking, seniority, anthropometric and physiological characteristics

## Abstract

**Background:** Little is known about the anthropometric and physiological profiles of lower-ranking athletes who aspire to rise to the pinnacle of their profession.

**Aim:** The aim of this study was to create anthropometric and physiological profiles of Hungarian male rowers of different age categories (15–16, 17–18, and over 18 years), sports rankings and career lengths.

**Materials and Methods:** Anthropometric and physiological profiles were created for 55 juniors, 52 older juniors and 23 seniors representing seven of the largest Hungarian rowing clubs. One-way independent analysis of variance (ANOVA) was used to compare arithmetic means.

**Results:** Rowers in older age categories were significantly taller (185.0 ± 5.0 cm vs. 183.0 ± 7.3 cm vs. 178.7 ± 7.2 cm) and heavier (81.1 ± 8.8 kg vs. 73.7 ± 8.4 kg vs. 66.8 ± 12.3 kg) than their younger peers, with significantly higher BMI values and larger body dimensions. Compared to younger athletes, rowers in older age categories also covered 2,000 m significantly faster (6.6 ± 0.3 min vs. 6.9 ± 0.4 min vs. 7.5 ± 0.5 min) while developing significantly more power (372.2 ± 53.0 W vs. 326.8 ± 54.5 W vs. 250.6 ± 44.6 W). Similarly, seniors and older juniors had higher values of maximal oxygen uptake and force max (by 6.2 and 7.0 ml/kg/min, and by 263.4 and 169.8 N). Within the older juniors, internationally ranked rowers had significantly greater body height (+ 5.9 cm), body mass (+ 6.1 kg), sitting height (+ 2.7 cm), arm span (+ 7.9 cm), limb length (+ 3.73 cm) and body surface area (+ 0.21 m^2^). They also rowed 2,000 m significantly faster (–0.43 min, *p* < 0.001) and had significantly higher values of power (+ 58.3 W), relative power (+ 0.41 W/kg), jump height (+ 4.5 cm), speed max (+ 0.18 m/s) and force max (+ 163.22 N).

**Conclusion:** The study demonstrated that potential differences in anthropometric and physiological profiles are more difficult to capture in non-elite rowers, and that the final outcome may be determined by external factors. Therefore, athletes with superior aptitude for rowing are more difficult to select from among lower-ranking rowers, and further research is needed to determine specific training requirements to achieve the maximum rowing performance.

## Introduction

Rowing is a sport discipline that has been extensively studied (Shephard, 1998). Penichet-Tomás et al. (2019) defines it as a cyclic sport with a strength endurance nature in which successful performance depends on technical (Baudouin and Hawkins, 2004; Shaharudin and Agrawał, 2016), anthropometric/biomechanical (Bourgois et al., 2000; Battista et al., 2007; Alacid et al., 2011; Forjasz, 2011; Almeida-Neto et al., 2020) and physiological characteristics (Messonier et al., 1997; Slater et al., 2005; Mikulić, 2008; Jürimäe et al., 2010; Majumdar et al., 2017; Maciejewski et al., 2019).

Performing simulations of official competitions can be of great value for evaluating and advancing athletes’ performance (Keenan et al., 2018; Penichet-Tomás et al., 2019). To improve rowing training methods and the selection of athletes with superior aptitude for the sport, it is useful to conduct both studies that assess the motor development of rowers and those that examine the relationship between rowers’ anthropometric and physiological characteristics and the results that they obtain (Koutedakis, 1989; Lawton et al., 2011). The anthropometric characteristics of athletes often reflect the physiological, functional, and biomechanical demands of their specific sport as well as modifications associated with training and diet (Battista et al., 2007). However, as Mikulić (2008) points out, some characteristics (e.g., anthropometric length and breadth measurements) are almost exclusively genetically determined and can be difficult to change *via* training. Therefore, precise information regarding the anthropometric and physiological status of rowers is a fundamental issue in contemporary rowing.

In sports demanding high force production, muscle mass may be closely associated with performance outcomes (Peterson et al., 2006; Kavvoura et al., 2018). In these sports in general and in Olympic rowing in particular, greater fat-free body mass may favor increased performance in competition (Schranz et al., 2010; Penichet-Tomás et al., 2019). In addition, given the importance of force production, anthropometric variables (i.e., body mass, body height, length of legs and body span) and muscular strength endurance of the trunk and upper and lower limbs are also associated with rowing performance (Majumdar et al., 2017; Maciejewski et al., 2019).

Reports on the anthropometric characteristics of adult rowers (females and males) stress the importance of body mass (Secher and Vaage, 1983; Bourgois et al., 2000; Forjasz, 2011; Giroux et al., 2017; Maciejewski et al., 2019) and body size and proportions (Hebbelinck et al., 1980; Mikulić, 2008; Schranz et al., 2010; Majumdar et al., 2017; Penichet-Tomás et al., 2019) as determinants of success in rowing at the international level. A comparative study of male and female fixed-seat rowers revealed that body height was the best predictor of performance in male rowers, and muscle mass—in female rowers (Penichet-Tomas et al., 2021). This observation could suggest that high lean body mass and a favorable power-to-body mass ratio are better predictors of success than high body mass because increased body mass and BMI negatively impacted on career attainment (Winkert et al., 2019).

More detailed analyses have also taken other factors into account in relation to anthropometric and physiological characteristics and performances in motor tests and sport competitions. These factors include different rowing modalities, such as sliding seat or fixed seat rowing (Penichet-Tomás et al., 2019); different boat types, i.e., sweeping or sculling (Claessens et al., 2005); events, e.g., single scull, skiff, or coxless pair (De Larochelambert et al., 2020); position occupied in the boat (Lawton et al., 2011); weight category, i.e., heavyweight or lightweight (Steinacker, 1993); ranking, e.g., world and Olympic champions vs. club members and college/university rowers (Mikulić and Ružić, 2008); and age, e.g., juniors vs. older juniors vs. seniors (Mikulić, 2008). These analyses have shown that, in general, more successful rowers are typically taller and heavier than less successful ones (Bourgois et al., 2000). Junior rowers are generally similar to adult heavyweight rowers in stature, except that the juniors tend to be lighter (Mikulić, 2008). Physiologically speaking, elite rowers differ from their less successful peers in terms of a higher average VO_2max_, and they typically have better technique, with a more efficient recovery phase (particularly with regard to the timing of forces at the catch), a faster stroke rate and a stronger, more consistent and effective propulsive stroke (Hagerman, 1984; Smith and Spinks, 1995; Hofmijster et al., 2007; Lawton et al., 2011). If all other factors are equal, rowers who can maintain greater net propulsive forces will achieve faster boat speeds (Smith and Spinks, 1995; Lawton et al., 2011).

In addition to size and physique, relative body proportions are important in rowing, in particular relative arm length and leg length (Claessens et al., 2005). A report comparing medalists and non-medalists at world championships indicated that the more successful lightweight rowers were more mesomorphic and less endomorphic and tended to have a shorter sitting height and longer upper and lower extremities (Rodriguez, 1986), which increase biomechanical efficiency. These findings are consistent with those of reports on heavyweight rowers (Kleshnev and Kleshnev, 1998; Shephard, 1998). Proportionally longer arms and legs not only correspond to a larger size but also give sweep rowers a biomechanical advantage due to the increased length of the levers (Piotrowski et al., 1992; Skład et al., 1993, 1994; Claessens et al., 2005; Penichet-Tomás et al., 2019). The best younger oarsmen also tend to be taller and heavier and have greater length, breadth and girth than their less successful peers (Bourgois et al., 2000; Mikulić, 2008; Forjasz, 2011).

A 2,000 m Olympic rowing competition requires a mixture of aerobic and anaerobic power (Wolf, 2016). These events require maximum exertion for a duration of five to 7 min (Steinacker et al., 1986). During this time, the relative anaerobic contribution ranges from 21 to 30% (Secher, 1993), which means that, in addition to a large aerobic capacity, a highly developed anaerobic capacity is also essential for successful international performance (Hagerman, 1984; Mäestu et al., 2005).

In general, rowing imposes heavy physiological demands, requiring a high degree of power and endurance, and a successful performance also requires a high level of technical proficiency (Secher, 1990; Keenan et al., 2018). According to Jurišić et al. (2014), aerobic metabolism provides 75–80% of the energetic demands during a rowing competition. Thus, as would be expected, elite rowers display impressive aerobic capacities: the VO_2max_ of internationally successful rowers regularly exceeds 5 l/min, it exceeds 6.0 l/min fairly often, and it sometimes reaches or exceeds 6.5–7.0 l/min at ventilation values above 240 l/min (Secher, 1993; Steinacker, 1993; Jurišić et al., 2014).

In sliding-seat Olympic rowing, around 75–80% of the power produced by successful elite rowers during a rowing stroke comes from their legs, and around 20–25% from their arms (Cosgrove et al., 1999). Various researchers have mentioned the ability of rowers to tolerate relatively high lactic acid (LA) concentrations during both rowing (Steinacker et al., 1999; Jürimäe et al., 2000) and leg press exercises that were conducted at individual physical working capacity (PWC) (calculated as heart rate (HR) 205–1/2 age) (Jürimäe et al., 2010). During the leg press exercise test, the subjects achieved a mean of 113.4 ± 38.5 repetitions with a mean duration of 450.2 ± 99.1 s, a mean HR of 137.4 ± 14.2 beats min^–1^, and a mean LA concentration of 7.62 ± 2.83 mmol l^–1^. The practical significance of these findings is that rowing exercise should stimulate increased oxygen uptake and raise the threshold (in terms of percentage of maximum oxygen uptake) at which blood LA concentration begins to increase substantially (Jurišić et al., 2014).

A review of the literature indicates that most published studies described the profiles of highly successful athletes and/or compared them with the profiles of lower-ranking rowers. Meanwhile, variables such as age category, ranking and length of the sports career have been explored by very few researchers. Moreover, there is a general scarcity of studies that approach the subject in a comprehensive manner and analyze intermediate rowers who have not yet achieved international success. Therefore, it remains unknown whether endogenous factors (anthropometric and physiological characteristics) determine the success of intermediate level athletes, or whether other external factors (such as organizational factors) also play a role. Trainers working with intermediate rowers could find it more difficult to capture minor differences in the anthropometric and physiological profiles of athletes that differ in ranking and career length. As a result, the elements of the training program may not be adapted to specific training goals, which can undermine the program’s effectiveness. It should also be noted that the number of athletes characterized by lower rowing performance is much higher than the number of elite athletes who win the most prestigious rowing championships. Hungarian rowers belong to the latter group. Only one Hungarian rower qualified for the Tokyo 2021 Olympic Games in Tokyo, and he ultimately came tenth in the men’s single scull category. Therefore, this study had two objectives: (a) to develop anthropometric and physiological profiles of Hungarian male rowers belonging to different age categories (15–16, 17–18, and over 18 years), had different sports rankings (international vs. club) and different career lengths (seniority levels); (b) to identify and explain potential differences between the analyzed groups of athletes who do not represent the highest level of rowing performance.

## Materials and Methods

### Participants

The study was conducted in Gyor rowing club, and the sample consisted of 130 male rowers from the seven largest Hungarian rowing clubs. The study lasted for three consecutive days in the middle of the racing season (8 days after one rowing regatta and 7 days before the next rowing regatta). The participants were selected by targeted sampling (based on the researchers’ arbitrary decision), and all rowers from the seven clubs were analyzed in the sampling process. The participants differed in ranking and length of sports career. Each rower was assigned to one of the three age categories: juniors (*N* = 55, range: 15–16 years), older juniors (*N* = 52, range 17–18 years), and seniors (*N* = 23; over 18 years). The senior group was relatively young, and the oldest senior rower was only 22. The following inclusion criteria were applied in the targeted sampling procedure: rowers in all age groups had to hold a valid competition license and participate in national and/or international competitions for minimum 1 year. All rowers had valid medical certificates; they participated regularly in training, and they did not limit their physical activity levels (for whatever reason) to the extent that could significantly affect their motor fitness. The training program was consistent with the guidelines of the Hungarian Rowing Federation Training Plan: 12–13 h/week for 15- to 16-year-olds, 14–15 h/week for 17- to 18-year-olds, and 16–17 h/week for 19- to 22-year-olds. The aerobic-to-anaerobic training ratio in the above groups was 80:20%, 75:25 and 70:30%, respectively. Athletes with an international ranking participated in training camps organized by the Hungarian Rowing Federation two to three times a year (depending on age group). It was hypothesized that the anthropometric and physiological characteristics of the rowers, as well their performance while rowing a 2,000 m distance and on motor tests would differ depending on their age, ranking, and length of sports career.

This research was conducted in line with the guidelines and policies of the Health Science Council, Scientific and Research Ethics Committee (IV/3067-3/2021/EKU), Hungary, and in accordance with the Declaration of Helsinki. Each participant was provided with detailed information about the purpose of the study, potential risks, measurement methods, and the techniques in motor tests that could be practiced during training sessions held directly before the study. All rowers gave voluntary informed consent to participate in the study by signing consent forms.

### Procedures, Data Collection and Equipment

Each rower was subjected to anthropometric and physiological tests in the middle of the 2020 racing season. On day one, anthropometric features were measured, on day two, the athletes performed motor tests, and on day three, they covered a distance of 2,000 m.

The coaches in charge of the rowers in the sports clubs helped us with the measurements. At all times, the coaches were instructed not to engage the subjects in any strenuous training the day before the testing took place. Each subject was always tested in the morning and the participants were instructed to eat a light meal (800–1,200 kcals) containing mainly carbohydrates (60–70%) not later than 3–4 h before the study (Williams, 1999). Body height was measured to the nearest 1 mm with a calibrated Soehlne Electronic Height Rod 5003 (Soehlne Professional, Germany) according to standardized guidelines. Body mass (measured to the nearest 0.1 kg), BMI and body composition characteristics, such as body fat percentage (BFP) and skeletal muscle mass (SMM), were determined by bioelectrical impedance with an InBody 720 body composition analyzer. The remaining anthropometric characteristics, such as sitting height [cm], arm span [cm], limb length [cm] and BSA [m^2^], were measured with the use of the Weiner and Lourie (1969). Skin fold measurements (biceps, triceps, scapula, suprailiac, abdomen, thigh, lower leg) were obtained using a Harpenden caliper.

#### Estimation of Relative Body Fat Content

The calipermetric estimation of relative body fat content that was developed by Parízková (1961) was used. This procedure requires the measurement of five skinfold thicknesses: over the biceps and triceps, subscapular, suprailiac and medial calf. The sum of the five skinfold values is multiplied by 2; the product is then used to look up the estimated relative body fat content in a table.

#### Countermovement Jumping

The power output of the lower extremities and the height attained by the center of body mass during vertical jumps were measured with a PJS-4P60S force plate (“JBA” Zb. Staniak, Poland) with a 400 Hz sampling rate (Gajewski et al., 2018; Batra et al., 2021). The force plate was connected *via* an analog-to-digital converter to a PC with MVJ v.3.4 software (“JBA” Zb. Staniak, Poland). The amplifier was connected to a PC *via* an A/D converter. Measurements were performed using the MVJ v. 3.4 software package (“JBA” Zb. Staniak, Poland). In the physical model that was used for calculations, the subject’s body mass was treated as a point affected by the vertical components of external forces: the force of gravity acting on the body and the vertical component of the platform’s reactive force. Each subject performed three counter-movement jumps (CMJ) with maximal force. A CMJ is a vertical jump from a standing erect position, preceded by a counter- movement of the upper limbs and lowering of the body mass center before take-off. Each subject was asked to perform a countermovement jump from the force plate to determine maximal force [N] and the rate of displacement [m/s]. From these measurements, jump height (by integrating ground reaction forces) [cm] and peak power [W] were determined. Using the body mass of the subject, the relative peak power [W/kg] was calculated.

#### 2,000 m Maximal Rowing Ergometer Test

The participants were asked to perform an all-out 2,000 m test a certified rowing ergometer (Concept 2 D-model). The screen of the ergometer was set to display the number of meters remaining, the average 500 m time and the accumulated time.

The power output in watts (W) was measured over 2,000 m. The calculation of watts was performed as follows: First, the distance was defined: distance = (time/number of strokes) × 500. In the next step, the concept of a “split” was clarified: split = 500 × (time/distance). The watts were calculated as 2.8/(split/500). There were slight differences in intensity due to individual changes in stroke value and ability to keep the 500 m split time constant. Prior to all tests, each participant warmed up for 6 min on a 500 m distance. Participants then rested for 6 min, during which time they performed stretching exercises. The estimated relative aerobic capacity (ErVO_2_) was calculated by using the formula of McArdle et al. (2007) for men: ErVO2 = (Y × 1000)/BM, where BM is body mass, and Y = [BM < 75 kg; 15.1− (1.5 × time)]; BM = > 75 kg; 15.7− (1.5 × time)]. The power delivered over 2,000 m was divided by body weight to obtain the relative performance (rW 2k).

Due to time and logistical constraints, including the need to perform a relatively high number of separate measurements within three consecutive days in a specific club to minimize disturbances to the athletes’ training and changes in their condition, this study did not examine heart rates (HR) and all indicators of acid-base balance, such as the concentration of lactic acid in the blood, alkaline deficiency or excess, blood pH and current molecular pressure of CO_2_.

### Statistical Analysis

Measurements were statistically processed with Statistica PL, v. 13.5. Based on the median length of participation in rowing competitions (juniors, 3 years; older juniors, 5 years; seniors, 7 years), the athletes in each age category were further divided into two subcategories: greater and lesser seniority. The rowers were ranked as international (participants in international competitions) or club (participants in inter-club competitions at the national level) level. Normality was verified with the Shapiro–Wilk test. It was checked that all tested features have normal distributions. Therefore, for comparisons of two arithmetic means, Student’s *t*-test was used. To compare three arithmetic means, one-way analysis of variance (ANOVA) was used. If ANOVA indicated a significant difference, Tukey’s Honestly Significant Difference (HSD) test was used for *post hoc* analysis. Cohen’s d was used as a measure of the effect size of differences between male and female rowers, and it interpreted according to the modified thresholds (Cohen, 1988) for sports sciences (Hopkins, 2016) as trivial (0.2), small (0.21–0.6), moderate (0.61–1.2), large (1.2–1.99) and very large (>2.0). Statistical significance was set at *p* ≤ 0.05.

## Results

### Analysis 1: Anthropometric and Physiological Characteristics

Table 1 presents the anthropometric characteristics, body composition, motor performance and physiological characteristics of the male rowers in the following age categories: juniors (15–16 years), older juniors (17–18 years) and seniors (18–22 years). Senior and older junior rowers were significantly larger than junior rowers in terms of height and body mass (height: + 6.25 and 4.32 cm, respectively; body mass: 14.32 and 6.88 kg; *p*-values for all comparisons are given in Table 1) with a moderate to large effect size. Seniors were also significantly heavier than older juniors (+ 7.44 kg; *p* = 0.011) with a moderate effect size. Regarding BFP, seniors had a significantly higher value than juniors and older juniors (+ 4.28 and + 3.83%, respectively; *p* = 0.006 and *p* = 0.014, respectively) with a moderate effect size. FFM did not differ significantly between these groups. Although older groups had significantly higher BMI than younger groups, the values of all groups were within the norms (20.8–23.72 kg/m^2^) with a moderate to large effect size. Sitting height and arm span were significantly less in the youngest group than in the other two groups (for both comparisons, *p* < 0.001), but these measurements did not differ significantly between the older juniors and the seniors. Body surface area was also significantly larger in older groups than in younger groups (*p*-values ranged from *p* = 0.017 to *p* < 0.001), and the effect size ranged from 0.8 to 1.3, but the groups did not differ significantly in terms of limb length, skin fold thickness or body fat measured by Pařízková’s formula.

**TABLE 1:** Comparison of arithmetic means of men’s anthropometric, physiological and motoric parameters depending on the age categories.

Characteristics		Age category [years]									Difference		HSD *p*-value (*post hoc*)			Cohen’s *d*		
		15–16 (*N* = 55)			17–18 (*N* = 52)			19–22 (*N* = 23)										
			
		Mean	SD	Min-max	Mean	SD	Min-max	Mean	SD	Min-max	*F*	*p*	1–2	2–3	1–3	1–2	2–3	1–3
Body height [cm]		178.70	7.22	162.1–193.4	183.02	7.27	167.7–197.4	184.96	4.98	174.4–194.0	8.66	<0.001	0.004	*ns*	<0.001	0.66	0.16	1.11
Body mass [kg]		66.82	12.27	39.6–115.0	73.70	8.43	56.6–89.7	81.14	8.80	62.1–100.0	16.72	<0.001	0.002	0.011	<0.001	0.66	0.86	1.34
Body fat [%]		12.39	5.54	4.0–28.9	12.84	5.39	5.3–33.0	16.67	4.33	9.4–22.9	5.38	0.006	*ns*	0.014	0.006	0.07	0.84	0.79
Skeletal muscle mass [%]		41.90	5.04	14.1–52.6	43.30	3.47	27.2–49.2	41.12	3.83	29.9–46.6	2.50	*ns*	*ns*	*ns*	*ns*	0.32	0.60	0.42
BMI [kg/m^2^]		20.82	2.97	15.02–31.00	21.98	2.10	18.28–29.47	23.72	2.43	18.28–27.88	10.63	<0.001	0.049	0.018	<0.001	0.42	0.64	0.90
Sitting height [cm]		92.50	4.60	79.4–100.3	95.33	3.56	87.5–105.1	96.56	2.04	93.9–100.1	11.87	<0.001	<0.001	*ns*	<0.001	0.67	0.40	1.00
Arm span [cm]		181.13	13.00	104.3–196.0	188.43	8.56	168.5–203.0	189.29	5.53	179.7–197.5	8.58	<0.001	<0.001	*ns*	0.004	0.66	0.12	0.71
Limb length [cm]		101.01	4.07	92.1–111.0	102.40	4.98	90.3–111.4	103.02	3.98	96.5–113.8	2.14	*ns*	*ns*	*ns*	*ns*	0.29	0.13	0.20
BSA [m^2^]		1.67	0.36	0.89–3.08	1.88	0.26	1.32–2.31	2.09	0.25	1.50–2.63	16.87	<0.001	<0.001	0.017	<0.001	0.69	0.80	1.25
Skin fold thickness [mm]	*B* *i* *c* *e* *p* *s*	7.04	3.57	2–20	5.69	3.13	3–21	5.96	2.69	3–12	2.44	*ns*	*ns*	*ns*	*ns*	0.39	0.08	0.32
	*T* *r* *i* *c* *e* *p* *s*	14.16	5.66	5–29	12.08	4.50	5–26	12.65	4.57	5–20	2.36	*ns*	*ns*	*ns*	*ns*	0.39	0.12	0.28
	*S* *c* *a* *p* *u* *l* *a*	10.96	4.57	4–31	9.96	3.16	6–23	10.70	3.48	3–18	0.91	*ns*	*ns*	*ns*	*ns*	0.37	0.11	0.35
	*S* *u* *p* *r* *a* *i* *l* *i* *a* *c*	9.95	5.66	4–33	8.26	3.76	4–21	8.52	2.29	5–13	2.04	*ns*	*ns*	*ns*	*ns*	0.40	0.20	0.26
	*A* *b* *d* *o* *m* *e* *n*	14.06	6.89	5-42	12.29	4.81	5-26	12.48	3.82	7-22	1.44	*ns*	*ns*	*ns*	*ns*	0.28	1.12	0.30
	*T* *h* *i* *g* *h*	20.36	7.84	6-46	18.45	7.54	7-39	18.48	6.01	4-29	1.04	*ns*	*ns*	*ns*	*ns*	0.31	0.14	0.27
	*L* *o* *w* *e* *r* *l* *e* *g*	14.07	6.27	4-30	12.31	5.99	5-30	13.39	4.46	4-22	1.18	*ns*	*ns*	*ns*	*ns*	0.29	0.18	0.31
Body fat [%] *)		23.03	4.04	13.8-31.5	21.86	4.14	14.5-30.9	22.43	3.62	12.2-26.6	1.13	*ns*	*ns*	*ns*	*ns*	0.22	0.14	0.15
Peak power [W]		250.55	44.60	138-322	326.80	54.48	210-435	372.22	52.96	292-461	54.99	<0.001	<0.001	0.002	<0.001	0.47	0.84	2.56
RPP [W/kg]		3.76	0.53	2.11-4.71	4.42	0.51	3.10-5.31	4.59	0.45	3.57-5.35	30.74	<0.001	<0.001	*ns*	<0.001	1.26	0.34	1.62
Time 2,000 m [min]		7.51	0.51	6.85–9.09	6.87	0.41	6.20–7.90	6.56	0.32	6.08–7.08	46.61	<0.001	<0.001	0.019	<0.001	1.35	0.79	2.02
ErVO_2_ max [mL/kg/min]		66.43	9.49	38.32–82.47	73.44	6.31	56.69–88.19	72.61	5.59	60.76–84.99	11.59	<0.001	<0.001	*ns*	0.005	0.87	0.75	0.54
Jump height [cm]		36.02	4.97	23.5–44.4	40.59	7.62	24.7–58.9	38.44	6.31	22.9–51.0	6.86	0.001	<0.001	*ns*	*ns*	0.71	0.30	0.54
Speed max [m/s]		2.59	0.19	2.06–2.91	2.74	0.29	1.97–3.33	2.66	0.24	2.08–3.09	5.43	0.005	0.003	*ns*	*ns*	0.40	0.14	0.34
Force max [N]		1, 551.35	323.58	899–2,317	1, 721.19	283.77	1,180–2,712	1,814.74	272.22	1,328–2,548	7.75	<0.001	0.009	*ns*	0.001	0.55	0.33	0.84
RPM [W/kg]		48.43	5.69	34.8–60.9	52.41	7.88	30.2–63.4	49.39	6.04	36.3–63.2	4.90	0.009	0.006	*ns*	*ns*	0.58	0.40	0.17

*) Pařízková’s formula; *ns*, not statistically significant; RPP, relative peak power; RPM, relative maximal power; ErVO_2_, estimated relative maximal aerobic capacity; Cohen’s *d*, effect size.

The seniors covered the 2,000 m distance in a significantly shorter time than the older juniors and juniors (respective differences: 0.31 min and 0.95 min; *p* = 0.019 and *p* < 0.001; *d* = 0.8 and *d* = 2.0), and older juniors covered this distance 0.64 min faster than juniors (*p* < 0.001) with a large effect size (*d* = 1.4). The peak power that was generated also differed significantly between these groups: seniors generated 45.4 W more than older juniors and 121.7 W more than juniors (*p* = 0.002 and *p* < 0.001; *d* = 0.8 and *d* = 2.6, respectively); older juniors surpassed juniors by 76.3 W (*p* < 0.001). Senior and older junior rowers also had significantly higher maximal oxygen uptake than juniors (by 6.2 and 7.0 ml/kg/min; *p* = 0.002 and *p* < 0.001; *d* = 0.5 and *d* = 0.9, respectively) and force max (by 263.4 and 169.8 N; *p* < 0.001 and *p* = 0.009; respectively). In terms of jump height, speed max and relative peak power (RPM), seniors and older juniors did not differ significantly, but these values were significantly lower in the juniors than in the older juniors (by 4.57 cm, 0.15 m/s, 3.98 W/kg; *p* < 0.001, *p* = 0.003 and *p* = 0.006; *d* = 0.8 and *d* = 0.55, respectively).

### Analysis 2: Ranking of Rowers

In the groups of older juniors and juniors, significant differences in the studied characteristics were associated with differences in ranking (club vs. international). The older juniors had the larger number of significant differences between these ranking categories (Table 2). In this age group, the internationally ranked rowers were significantly taller (+ 5.88 cm, *p* = 0.0027) and heavier (+ 6.1 kg, *p* = 0.0078), and they had a longer sitting height (+ 2.67 cm, *p* = 0.0058), arm span (+ 7.90 cm, *p* = 0.0051), limb length (+ 3.73 cm, *p* = 0.0058), and BSA (+ 0.21 m^2^, *p* = 0.0028). Rowers in the older junior category with an international ranking were significantly taller (+ 5.88 cm, *p* = 0.0027) and heavier (+ 6.1 kg, *p* = 0.0078) than their club level peers, and they had a longer sitting height (+ 2.67 cm, *p* = 0.0058), arm span (+ 7.90 cm, *p* = 0.0051), and limb length (+ 3.73 cm, *p* = 0.0058), as well as a larger BSA (+ 0.21 m^2^, *p* = 0.0028) with a moderate to large effect size. In addition, they covered the 2,000 m distance in significantly less time (−0.429 min, *p* < 0.0001) and developed greater peak power (+ 58.3 W, *p* < 0.0001) and relative peak power (+ 0.41 W/kg, *p* = 0.0037) with a moderate to large effect size. In motor tests, they obtained higher jump height (+ 4.5 cm, *p* = 0.0325), speed max (+ 0.178 m/s, *p* = 0.0255), and force max (+ 163.22 N, *p* = 0.0373) with a moderate effect size.

**TABLE 2:** Comparison of arithmetic means of older junior male rowers anthropometric, physiological and motoric parameters depending on the ranking categories.

Characteristics	Ranking category				Difference		Cohen’s *d*
	International (*N* = 24)		Club (*N* = 28)				
		
	Mean	SD	Mean	SD	*t*	*p*	
Body height [cm]	186.18	5.23	180.30	7.74	3.15	0.003	0.97
Body mass [kg]	76.98	6.23	70.88	9.13	2.77	0.008	0.79
Sitting height [cm]	96.77	3.24	94.10	3.40	2.88	0.006	0.83
Arm span [cm]	192.68	6.04	184.78	8.79	3.71	0.001	1.19
Limb length [cm]	104.41	3.96	100.68	5.18	2.88	0.006	0.80
BSA [m^2^]	1.99	0.19	1.78	0.28	3.14	0.003	0.88
Peak power [W]	356.54	41.38	298.24	50.60	4.40	<0.001	1.26
RPP [W/kg]	4.63	0.42	4.22	0.52	3.05	0.004	0.87
Time 2,000 m [min]	6.65	0.27	7.08	0.41	–4.30	<0.001	1.24
Jump height [cm]	43.01	6.04	38.51	8.31	2.20	0.033	0.62
Speed max [m/s]	2.84	0.21	2.66	0.32	2.30	0.026	0.67
Force max [N]	1, 809.08	249.97	1, 645.86	293.47	2.14	0.037	0.61

Regarding the juniors, two of their characteristics differed significantly between the ranking categories: international level juniors achieved higher power (+ 29.91 W, *p* = 0.0274) and covered 2,000 m in a shorter time (−0.342 min, *p* = 0.0271). These groups also differed in terms of some other characteristics, although the differences were not statistically significant.

### Analysis 3: Length of Rowers’ Sports Careers

In all age categories, the length of the athletes’ sports career was not associated with significant differences in anthropometric characteristics, body components, results of motor performance tests, and time to complete a 2,000 m distance (*p* > 0.05) with a trivial to small effect size. The only exception was the level of adipose tissue in lower leg skin, which was significantly higher (difference: + 3.48 mm, *p* = 0.044) in the group of rowers that had competed for a shorter time, which is probably due to chance. Interestingly, however, even though the differences were not statistically significant, senior and older junior rowers tended to have higher body mass (+ 1.40 kg and + 2.68 kg, respectively), fat percentage (+1.86% and +0.71%, respectively), and BMI (+ 0.61 kg/m^2^ and 1.45 kg/m^2^, respectively).

## Discussion

### Anthropometric and Body Composition Profiles

Rowers in specific age categories were assessed in terms of skeletal structure (body height, body mass, sitting height, arm span, limb length, BMI, BSA), body composition (BFP, SMM) and thickness of skin folds (biceps, triceps, scapula, suprailiac, abdomen, thigh, lower leg). It was found that rowers in older age categories had higher body mass, BMI, and BSA than their younger peers. Compared to the juniors, seniors and older juniors had greater body height, sitting height, and arm span. These results are similar to those of a study of Croatian rowers by Mikulić (2008). In that study, Croatian champions and members of the Croatian national team were classified into elite seniors (28.1 ± 3.0 years), sub-elite juniors (22.16 ± 2.8 years), and elite juniors (17.6 ± 0.4 years). They found that the elite seniors were taller and heavier than the sub-elite juniors (+ 5.4 cm, 4.3 kg) and the juniors (+ 5.1 cm, + 11.1 kg).

Rowers with larger body dimensions (body mass, body height, length of lower and upper extremities) achieve proportionally better rowing performances (Cosgrove et al., 1999; Yoshiga and Higuchi, 2003; Mikulić, 2008). This is probably the reason why, in the present study, the seniors and older juniors, who had larger body dimensions than the juniors, covered 2,000 m faster and developed more peak power, RPP and force max over this distance, while achieving a higher ErVO_2max_. However, with regard to jump height, speed max, and RPM, only the older juniors differed significantly from the juniors.

Hungarian rowers in the senior age group had significantly higher values of BFP than older juniors and juniors. A similar phenomenon was observed in Croatian elite rowers, where BFP was lower in the juniors than in the elite and sub-elite seniors, although no difference was observed between the two groups of seniors (Mikulić, 2008). Similar differences were found in Belgian rowers: compared to non-finalists, finalists were heavier and taller with greater length, breadth (expect for the bicristal diameter), and girth (Bourgois et al., 2000).

In the present study, many of the differences in anthropometric characteristics between the international and club level rowers were not statistically significant. These results are properly interpreted as inconclusive, as explained by the guidelines of the American Statistical Association (Wasserstein and Lazar, 2016) and many other experts (e.g., Greenland et al., 2016; Amrhein et al., 2017). Therefore, the present results are not necessarily in disagreement with the findings of Secher (1975), who found that the body mass of internationally competitive rowers was greater than that of club rowers, or the results of Penichet-Tomás et al. (2019), who reported that higher-performing rowers had significantly larger anthropomorphic measurements than lower-performing ones. Our assumptions were confirmed by the fact that all of the examined rowers were only aspiring to become elite performers, and none of them was a finalist of prestigious rowing regattas. In addition, the senior group was relatively young (19–22 years), whereas the average age of male and female single scullers in the Olympic finals has risen by roughly 7 years, from around 24–31 (World Rowing, 2015). The above can be attributed to the fact that elite rowers’ efficiency remained stable because their oxygen uptake at 300 W was similar at the ages of 25 and 31. Some elite rowers, such as Steven Redgrave and Eskild Ebbesen, won their last Olympic titles at the age of 38 and 40, respectively (Nybo et al., 2014). Thus, it would be interesting to perform another study with a different sample of Hungarian rowers or measure the same individuals in the following years to see if the differences between the groups being compared would continue to be statistically non-significant when taking rankings and length of sporting career into account. The only exception was the group of older juniors, where significant differences in anthropometric and physiological characteristics were noted between rowers with international and club rankings, and where the studied parameters were more favorable in the former category of athletes. There are several reasons why older juniors with an international ranking had an advantage over their peers with a club ranking. Firstly, these athletes had participated in the highest number of training camps (qualification rounds) during the selection of the Hungarian national team. Secondly, they were most successful in rowing events, both in terms of the scored results and the rank of rowing regattas.

The mean body mass of the senior rowers in this study (81.14 kg) is similar to that of the elite Olympic rowers measured by Forjasz (2011), which ranged from 80 to 85 kg. The Hungarian senior rowers in the present study had a mean height of 184.96 ± 4.98 cm, which is also very similar to what Forjasz (2011) measured.

In the present study, the differences in FFM between age categories were not statistically conclusive. However, Mikulić (2008) found that FFM was greater in elite seniors than in elite juniors (+ 6.1 kg). Moreover, rowing performance has generally been found to correspond closely to FFM values (Cosgrove et al., 1999; Yoshiga et al., 2000), and FFM is considered one of the best predictors of performance (Ingham et al., 2002; Riechman et al., 2002). Thus, it would be interesting to investigate the FFM of Hungarian rowers with a different sample, and perhaps combine those results with the results of this study *via* statistical meta-analysis.

For rowers, BMI values of approx. 24 kg/m^2^ are considered optimal olympic rowing (Barrett and Manning, 2004; Claessens et al., 2005; Sanada et al., 2009), but the BMI of traditional rowers is sometimes higher (Penichet-Tomas et al., 2021). The mean BMI value of the elite seniors in the present study (23.72 kg/m^2^) was very close to this benchmark, while those of all the other rowers were within normal limits (range: 20.82–23.72 kg/m^2^). However, when considering these results, it is important to remember that the BMI does not give reliable information about the body composition of sports athletes and does not allow an important distinction to be made between the distribution of fat and muscle tissue in the lower and upper half of the body (Garrido-Chamorro et al., 2009; Mazić et al., 2009).

### Physiological and Motor Performance Profile

The results of the study presented here support the conclusion of Jurišić et al. (2014) that aerobic metabolism predominantly determines success in a 2,000 m rowing race on a simulator. Although lactate anaerobic threshold was not assessed in this study, older rowers finished the 2,000 m simulation in significantly less time than their younger counterparts while attaining higher values of ErVO_2max_ and RPP. In addition, the older rowers developed significantly more power than their younger peers, and the seniors and older juniors developed significantly higher force max values than the juniors although the difference between the seniors and older juniors was not statistically significant.

The mean VO_2max_ values of the junior, older junior and senior Hungarian rowers examined in the present study were higher than those reported for Croatian rowers (Mikulić, 2008) (66.43, 73.44, and 72.61 mLmin^–1^kg^–1^ vs. 62.5, 55.3, and 58.4 mLmin^–1^kg^–1^, respectively). When combining all these results with those of a study of Croatian 12–13-year-old rowers (VO_2max_: 48.8 mLmin^–1^kg^–1^) (Mikulić and Ružić, 2008), it appears that there is a general trend of VO_2max_ values increasing during the early years of training when rowers are at younger ages. This increase could be due in part to the fact that the growth processes of men continue up to 21 years of age, and these processes contribute substantially to rowing performance (Almeida-Neto et al., 2020). However, an analysis of changes in the maximal oxygen uptake at certain ages of Polish male rowers showed substantial improvement at 19–19.9 and 21–22 years (Klusiewicz et al., 2014). Maximal oxygen uptake, which is the gold standard for cardiorespiratory fitness, is a multifactorial trait influenced by environmental factors (e.g., exercise training) and genetic factors (Rankinen, 2011; Mann et al., 2014; Williams et al., 2017). However, improvements in cardiorespiratory fitness in response to exercise training vary greatly between individuals, with some people responding well or very well (“responders” or “high-responders”) to exercise training, whereas others do not respond so well following similar exercise training (Mori et al., 2009; Bouchard et al., 2015; Williams et al., 2017).

Finally, it should be remembered that the differences in performance between these Hungarian, Croatian and Polish rowers may also be due to variations in the conditions in which they were tested. Visual and verbal feedback may be factors that can substantially improve rowing performance over 2,000 m (Stine et al., 2019).

In conclusion, these results for Hungarian rowers are in line with those of the previous studies cited in the introduction of this paper that suggest that a rower’s height and length are proportional to his/her level of rowing performance (e.g., Yoshiga and Higuchi, 2003; Mikulić, 2008). In the future, different age categories should be compared to optimize training outcomes in rowers. On the one hand, a clear improvement in the performance of Hungarian male rowers transitioning to an older age category could indicate that the selected training methods are adequate. However, minor differences in the anthropometric and physiological profiles of rowers with a different ranking and different career length could imply that the selection of training approaches is not optimal, which suggests that other measures are needed to fully tap the performers’ potential in this stage of the training process. For example, to achieve high-level rowing performance, the training program of rowers should include the development of strength and endurance capacity with the aim of increasing muscle mass, aerobic capacity and metabolic efficiency, and decreasing percent body fat (Lakomy and Lakomy, 1993; Warmenhoven et al., 2018; Durkalec-Michalski et al., 2019). Such activities are an indispensable part of the selection process, and they could be lacking among Hungarian rowers in the preparation process.

The lack of significant differences in individual age categories when variables such as ranking and competition seniority were taken into account stands in contrast to numerous studies conducted by other authors. However, these authors compared the finalists of major rowing events with intermediate rowers. Meanwhile, the examined group of Hungarian rowers had both club and international ranking, but the latter had not scored spectacular success in the international arena. As a result, the analyzed population was less diverse in terms of anthropometric and physiological profiles, and potential differences were much more difficult to capture. From the practical point of view, trainers may find it difficult to select the most promising rowers because the final result can be influenced by external factors (organizational, financial or motivational) that are not directly linked with endogenous factors (anthropometric and physiological characteristics).

In our opinion, this is one of the first studies to address this issue, but definitive conclusions cannot be drawn at this stage of research, which is why further studies of Hungarian rowers spanning a longer period of time are needed.

### Strengths and Limitations

This paper makes a novel contribution to the literature by providing information about the anthropometric and physiological characteristics of Hungarian rowers who are relatively young and are only aspiring to become elite athletes. This study makes the first ever attempt to capture differences in the anthropometric and physiological characteristics of intermediate rowers. In our opinion, the above fact is a definite strength of the study because the number of non-elite athletes significantly exceeds the number of rowing champions who constitute a relatively small group. This approach contributes to the novelty of our study, but our findings are difficult to compare with those reported by other authors due to the general lack of research addressing intermediate athletes. The size of the analyzed sample was relatively large in comparison with similar studies (Mikulić, 2008; Klusiewicz et al., 2014); therefore, the formulated conclusions can be viewed with a relatively high degree of confidence.

The fact that HR values (minimum, average and maximum) and lactate anaerobic threshold values could not be included in the study because measurements were performed within a timeframe of three consecutive days is a limitation of this study. Despite the above, the study generated valuable insights about differences between age categories, including measurements of VO_2max_ values which are considered the gold standard for cardiorespiratory fitness. To complement our findings, acid-base balance indicators, including blood pH, partial pressure of CO2 in arterial blood (pCO2), HCO_3_^–^ ion concentration, and alkaline deficiency or excess (BE), should be examined in the future. Repeated measurements involving the same athletes during different training periods could also generate interesting results.

## Conclusion

Hungarian rowers in older age categories have higher values of anthropometric and physiological characteristics than younger ones. Within the older juniors but not in the other age categories, these characteristics are significantly better in rowers with an international ranking than in those with a club ranking. Within these age categories, length of sports career was not associated with significant differences between rowers.

The study revealed that potential differences in anthropometric and physiological characteristics are more difficult to identify in rowers who are not elite athletes and differ in age, ranking and length of the sport career than when rowing champions are compared with the remaining, lower-ranking rowers. As demonstrated on the example of Hungarian rowers, the ultimate success of intermediate rowers is determined not only by endogenous factors associated with training and anthropometric and physiological characteristics, but also by external factors (organizational, financial, and motivational). Further research is needed to confirm the present findings. For instance, it would be interesting to investigate whether athletes with optimal training conditions are more successful than those with less favorable training conditions. Future studies should also involve advanced statistical analyses, such as partial correlation analysis, to identify variables that exert the greatest influence on rowers’ performance. It would be interesting to perform a longitudinal study that examines how these characteristics change or remain the same as the season progresses, or even over several years of the athletes’ sports careers. These repeated examinations could provide an opportunity to assess more accurately whether the relationships between anthropometric and physiological characteristics and the results obtained in motor tests should play an important role in the process of sports selection.

## Data Availability Statement

The Excel data used to support the findings of this study are restricted by the Ethics Committee of the University of Warmia and Mazury in Olsztyn (UWM), Poland in order to protect participants’ privacy. Data are available from RP, E-mail: podstawskirobert@gmail.com for researchers who meet the criteria for access to confidential data.

## Ethics Statement

The studies involving human participants were reviewed and approved by Health Science Council, Scientific and Research Ethics Committee (IV/3067-3/2021/EKU). Written informed consent to participate in this study was provided by the participants’ legal guardian/next of kin.

## Author Contributions

RP designed this study. ZA, FI, IS, and RP contributed to data collection. KB and RP analyzed and interpreted the data and drafted the final version. RP and FI drafted the primary manuscript and with the assistance of ZA. All authors critically reviewed and approved the manuscript prior to submission.

## Conflict of Interest

The authors declare that the research was conducted in the absence of any commercial or financial relationships that could be construed as a potential conflict of interest.

## Publisher’s Note

All claims expressed in this article are solely those of the authors and do not necessarily represent those of their affiliated organizations, or those of the publisher, the editors and the reviewers. Any product that may be evaluated in this article, or claim that may be made by its manufacturer, is not guaranteed or endorsed by the publisher.
